# Teriparatide in postmenopausal osteoporosis: uncovering novel insights into efficacy and safety compared to other treatments – a systematic review and meta-analysis

**DOI:** 10.1530/EOR-23-0205

**Published:** 2024-09-02

**Authors:** Djandan Tadum Arthur Vithran, Anko Elijah Essien, Masoud Rahmati, Michael Opoku, Dong Keon Yon, Guillermo F López Sánchez, Ai Koyanagi, Lee Smith, Jae Il Shin, Wenfeng Xiao, Shuguang Liu, Yusheng Li

**Affiliations:** 1 Department of Orthopedics, Xiangya Hospital, Central South University, Changsha, Hunan, China; 2 National Clinical Research Center for Geriatric Disorders, Xiangya Hospital, Central South University, Changsha, China; 3 Research Centre on Health Services and Quality of Life, Aix Marseille University, Marseille, France; 4 Department of Physical Education and Sport Sciences, Faculty of Literature and Human Sciences, Lorestan University, Khoramabad, Iran; 5 Department of Physical Education and Sport Sciences, Faculty of Literature and Humanities, Vali-E-Asr University of Rafsanjan, Rafsanjan, Iran; 6 Center for Digital Health, Medical Science Research Institute, Kyung Hee University Medical Center, Kyung Hee University College of Medicine, Seoul, Republic of Korea; 7 Department of Pediatrics, Kyung Hee University College of Medicine, Seoul, Republic of Korea; 8 Division of Preventive Medicine and Public Health, Department of Public Health Sciences, School of Medicine, University of Murcia, Murcia, Spain; 9 Research and Development Unit, Parc Sanitari Sant Joan de Deu, Barcelona, Spain; 10 Centre for Health, Performance, and Wellbeing, Anglia Ruskin University, Cambridge, UK; 11 Department of Pediatrics, Yonsei University College of Medicine, Seoul, Republic of Korea; 12 Department of Joint Surgery, Honghui Hospital, Xi’an Jiaotong University, Xi’an, Shaanxi, China

**Keywords:** fractures, parathyroid hormone, postmenopausal osteoporosis, systematic review, teriparatide

## Abstract

**Objective:**

- The aim of the study was to evaluate the efficacy and safety of teriparatide compared to other treatments for postmenopausal osteoporosis.

**Methods:**

- A review of studies from 2000 to January 2023 analyzed randomized controlled trials on postmenopausal women treated with teriparatide (PTH 1–34), comparing it to placebo or other osteoporosis treatments. The analysis focused on bone mineral density (BMD), bone turnover markers, and clinical outcomes, employing Review Manager 5.4.1 and the RoB 2 tool for bias assessment.

**Results:**

- Our analysis of 23 randomized controlled trials (RCTs) found that PTH (134) treatment significantly increased lumbar spine BMD (mean difference (MD) = 0.02, 95% CI: 0.01–0.03) and femoral neck BMD (MD = 0.01, 95% CI: 0.00–0.01). However, there were no significant changes in total hip and radial bone BMD among the 3536 and 2046 participants, respectively. We also found that PTH (1–34) increased P1NP in a larger cohort (*n* = 1415) when compared to osteocalcin (*n* = 206). Although the risk of adverse events increased (relative risk (RR) = 1.65, 95% CI: 1.32–2.07), the incidence of fractures decreased significantly (RR = 0.57, 95% CI: 0.45–0.072), with no significant difference observed in mortality rates between treatment and control groups.

**Conclusion:**

- Teriparatide improves lumbar spine and femoral neck BMD in postmenopausal women. Particularly notable is the novel finding regarding its effect on radius BMD, an area less explored in previous research. Despite an uptick in adverse events, the marked decrease in fracture incidence confirms its clinical utility for high-risk osteoporosis patients, highlighting the necessity for ongoing investigations into its full skeletal effects.

## Introduction

Osteoporosis is a critical global health issue, disproportionately affecting postmenopausal women by leading to diminished bone mass, compromised bone strength, and an elevated risk of fractures. The World Health Organization underscores this, noting that approximately 30% of women post menopause have osteoporosis, which underscores the urgent need for efficacious management strategies (1, 2). Postmenopausal Osteoporosis (PMO) primarily results from estrogen deficiency, leading to structural deterioration of bone tissue and an increased susceptibility to fractures. These fractures not only cause pain and deformities but also severe health complications and, in extreme cases, premature mortality (3).

Despite considerable advancements in therapeutic options, fractures remain alarmingly prevalent among those with PMO, particularly in areas rich in trabecular bone like the lumbar spine and femoral neck. Fractures at critical sites, such as the hip, carry significant morbidity and mortality risks (4, 5, 6, 7, 8). The therapeutic landscape for PMO, traditionally divided into antiresorptive and osteoanabolic drugs, has been notably enhanced by teriparatide, a synthetic parathyroid hormone analog recognized for its bone anabolic properties since its approval in 2003 (9, 10, 11, 12, 13, 14, 15, 16, 17, 18, 19).

Research has broadly documented teriparatide’s impacts on bone mineral density (BMD) and fracture healing across various skeletal sites (3, 6, 18, 19, 20, 21, 22, 23, 24, 25, 26, 27, 28, 29, 30, 31, 32, 33, 34, 35, 36, 37, 38, 39, 40, 41, 42, 43, 44, 45, 46, 47, 48). Nonetheless, the specific effects of teriparatide, particularly on radial BMD, have yet to be thoroughly explored. Although some studies have highlighted teriparatide’s potential in enhancing bone density and reducing fracture risks, the concrete evidence, especially from the limited two RCTs focusing on these effects, does not show statistically significant differences between treatment groups. This critical understanding gap emphasizes the need for more targeted research (25, 26, 27, 28, 29, 30, 31, 32, 33, 34).

The systematic review by Metcalf *et al.* investigated the effects of various PTH peptides (1–34 and 1–84) across multiple skeletal sites but was limited by heterogeneity, obscuring the distinct impacts of each peptide form (35). Our study aims to bridge this gap by focusing exclusively on applying PTH 1–34, particularly its effects on the radial bone, hip, and lumbar regions in postmenopausal women. Through this specialized approach, we strive to provide novel insights into PTH 1–34’s specific impact on radial BMD, setting our research apart from broader analyses and illuminating the peptide’s distinct advantages and challenges.

In pursuing this aim, our study employs a rigorous methodology, systematically reviewing and meta-analyzing high-quality RCTs. This approach is designed to elucidate the nuanced role of teriparatide in the clinical management of PMO, potentially reshaping therapeutic strategies.

Addressing the complex landscape of PMO management and teriparatide’s nuanced applications, this study poses a critical question: How does teriparatide’s efficacy and safety in enhancing bone mineral density and reducing fracture incidence, compare with other standard postmenopausal osteoporosis treatments, given its unique anabolic properties?

To address the identified research gap, our study considered several objectives: first, to evaluate teriparatide’s impact on BMD at the lumbar spine, femoral neck, and notably, the radial bone, identify site-specific effects, and assess its broader therapeutic potential; secondly, to critically compare teriparatide’s efficacy in reducing fracture incidence with that of placebo and other established osteoporosis medications, defining its clinical value for individuals at heightened risk of fractures; and finally, to thoroughly explore teriparatide’s safety profile, emphasizing on adverse events, thereby providing a balanced perspective on its therapeutic application.

By addressing these objectives, our study seeks to provide deep insights into teriparatide’s efficacy and safety, advance therapeutic strategies for managing postmenopausal osteoporosis, and enrich the knowledge available to the scientific and clinical communities.

## Materials and methods

This systematic review and meta-analysis followed the 2020 PRISMA guidelines (see Supplemental Materials, see section on supplementary materials given at the end of this article) (49). The protocol can be accessed through PROSPERO (see Supplemental Materials).

### Study Design

Quantifying the impact of the parathyroid hormone (PTH 1–34) on postmenopausal osteoporosis is the purpose of this meta-analysis, which includes publications published between 2000 and January 2023. The research plan consisted of the following sections: i) establishing the goals of the study and the criteria for selecting relevant materials; ii) using the stated search terms and search algorithms to search the most pertinent databases for the topic of interest and the pertinent literature; iii) identifying relevant studies by comparing them to predetermined inclusion/exclusion criteria; iv) carefully collecting the necessary information using the data extraction form; v) data input and analysis using Review Manager version, version 2 of Cochrane Collaboration risk-of-bias assessment tool for randomized trials and Stata 15.0; and finally, vi) developing conclusions and interpretations from the data.

### Search strategy

#### Inclusion criteria

Our study adopted a structured approach defining the inclusion criteria, utilizing the PICO framework to ensure a comprehensive and targeted analysis as follows: i) Population: Our focus was on postmenopausal women diagnosed with osteoporosis, aiming to understand the therapeutic impact of teriparatide across this specific demographic. ii) Intervention: The intervention of interest was the administration of single daily injections of teriparatide (PTH 1–34) to the treatment groups. iii) Comparator: Participants in comparator groups received various anti-osteoporosis medications or placebo, including risedronate, zoledronic acid, alendronate, salmon calcitonin, elcatonin, general antiresorptive drugs, placebo, denosumab, abaloparatide, and romosozumab. These comparators were selected to offer a broad perspective on teriparatide’s efficacy and safety relative to the standard treatments for postmenopausal osteoporosis. iv) Outcome: The primary outcomes assessed were the incidence of vertebral fractures and changes in BMD at critical anatomical sites (total hip, lumbar spine, radius, and femoral neck). Secondary outcomes included bone turnover markers (P1NP, CTx, and osteocalcin), adverse events, and mortality. v) Study type: Only randomized controlled trials (RCTs) involving postmenopausal women with osteoporosis treated with PTH 1–34, with outcomes as specified above.

#### Exclusion criteria

The criteria of exclusion were as follows: i) repeated publications; ii) literature unrelated to the study topic; iii) conference summary, reviews, and patents with no detailed data; iv) animal experiments; v) study subjects with non-osteoporotic fractures; or non-vertebral fractures; vi) study subjects with secondary osteoporosis, such as (GIOP, malignant tumor associated bone diseases, bone metastases); vii) study subjects participants with underlying diseases such as autoimmune diseases, inflammatory bowel diseases, malignant tumors, and hypogonadism; viii) non-therapeutic literature or group design; ix) only the abstract but no full-text report; x) literature with inappropriate clinical study design (retrospective clinical trials, non-randomized controlled studies, observational studies, etc.); xi) studies that have participants with less than 6 months of teriparatide treatment; and xii) literature with incomplete data.

### Search strategy

We conducted a comprehensive search for studies about treating postmenopausal osteoporosis with PTH 1–34 using a detailed plan suggested by the Cochrane Collaboration. We looked for articles using specific search terms like ‘Parathyroid Hormone’, ‘Osteoporosis, Postmenopausal’, ‘Teriparatide’, and ‘Randomised Controlled Trial’ from 2000 to January 2023. The search included databases such as Web of Science, Cochrane Library, Scopus, Embase, and several Chinese databases without limiting language. We carefully checked the full texts and references of the studies we found to gather information on using PTH 1–34 for postmenopausal osteoporosis.

### Literature selection

Two separate reviewers checked titles and abstracts to find relevant papers. Subsequently, the full-text articles meeting the inclusion criteria, including adverse events, patient demographics, medications, treatment protocols, duration of follow-up, BMD outcome measures, and adverse event incidence, were accessed for data extraction. When many studies reported the same data, the most thorough data were obtained, and the studies were credited under one research name. A third reviewer was consulted to reach a consensus if the two disagreed. Cochrane standards were used to evaluate included studies to examine selection, detection, attrition, performance, and reporting biases, categorized as low, uncertain, or high risk of bias for quality evaluation.

### Data extraction

The studies included have been considered for the extraction of data. This included general data such as the first author of the literature, time, sample size, basic population information fracture site, post-fracture treatment method, and characteristics such as drug intervention method, treatment initiation time, treatment duration, and outcome measures of the test group and control group. Results included fracture rate, BMD change, bone turnover, death, and incidence of adverse events.

### Quality evaluation of study quality

Two authors independently utilized the Cochrane Collaboration risk-of-bias assessment tool for randomized trials, specifically version 2 (RoB 2), to assess the quality of the study. Based on the criteria mentioned above, the studies included in this analysis were categorized as either ‘low risk’, ‘high risk’, or ‘some concerns’. The potential for bias in the included studies was visually depicted using Review Manager (v5.4.1) and Stata 15.0. Disputes about the adequacy of the studies were resolved via deliberation among the review’s authors until a consensus was achieved.

### Statistical analysis

Statistical analyses were performed using specialized software designed for systematic reviews, namely Review Manager 5.4.1 (RevMan 5.4) and Stata 15.0. To analyze count data, like the incidence of adverse events or the fracture rate, we employed odds ratios (ORs) or risk differences (RDs). In the case of continuous data, such as BMD score or bone turnover, the mean difference (MD) was employed. The *I* ^2^ index was utilized to quantify the level of heterogeneity, representing the proportion of variation in effect estimates attributed to heterogeneity rather than random chance. An *I*^2^ value exceeding 50% indicated significant heterogeneity. The Peto method for combining effect sizes was used when *I*^2^ was ≤ 50%, while the random-effects model DerSimonian–Laird calculation method was used when *I*^2^ was > 50%. The publication bias assessment was conducted by utilizing the Egger test. A two-sided *P* value less than 0.05 is considered statistical significance.

## Results

### Identification and selection of studies

**Figure 1:**
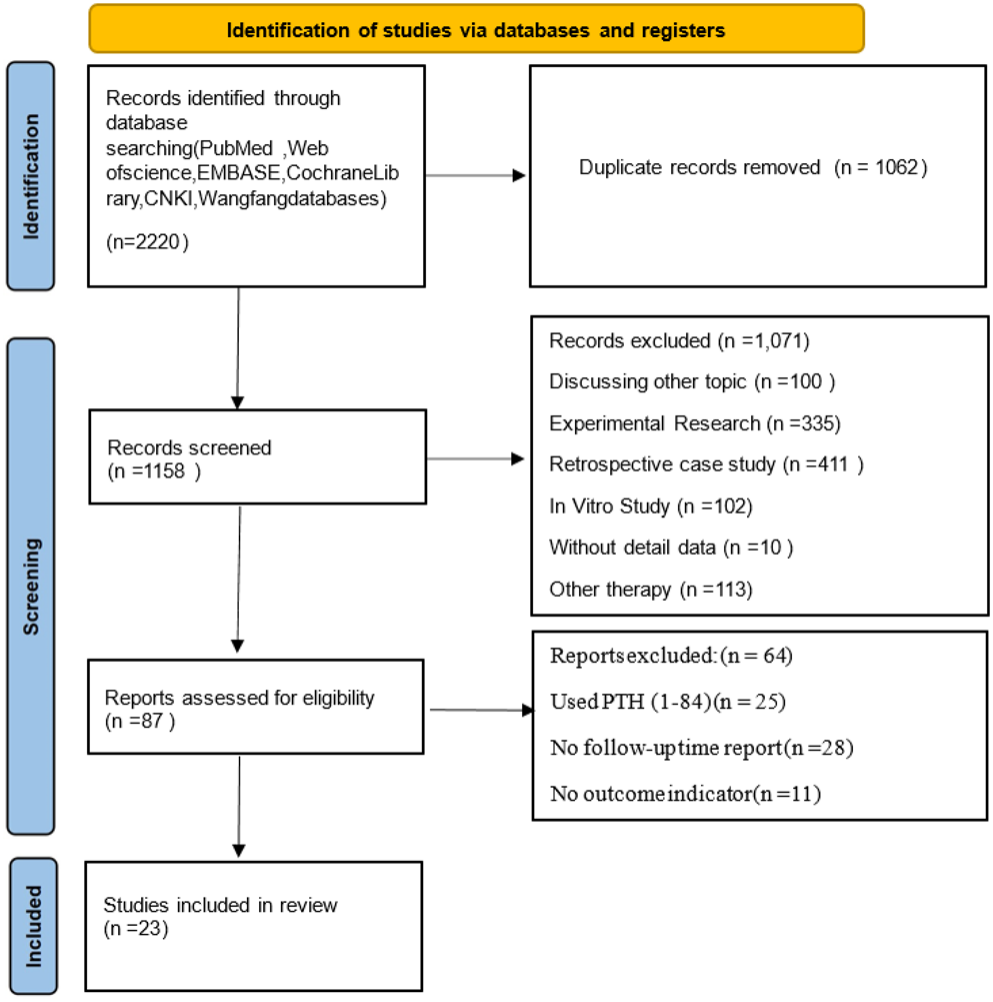
Literature screening flowchart.

In total, 2220 published articles were found through the search, but 1062 duplicate articles were removed. Of the 1158 remaining articles, 1071 were ineligible based on predetermined criteria. Of the remaining 87 full articles, 65 were excluded. In the end, the study evaluated 23 RCTs. No additional studies that met the established inclusion criteria were found by systematically examining the reference lists of the incorporated studies. The PRISMA statement’s flowchart (Fig. 1) illustrates the study’s screening and selection process. Supplementary Table 1 outlines the key features of these studies (See supplementary material). All studies involved patients randomly assigned to receive at least one daily dose of Teriparatide for at least 6 months.

#### Heterogeneity and publication bias

**Figure 2:**
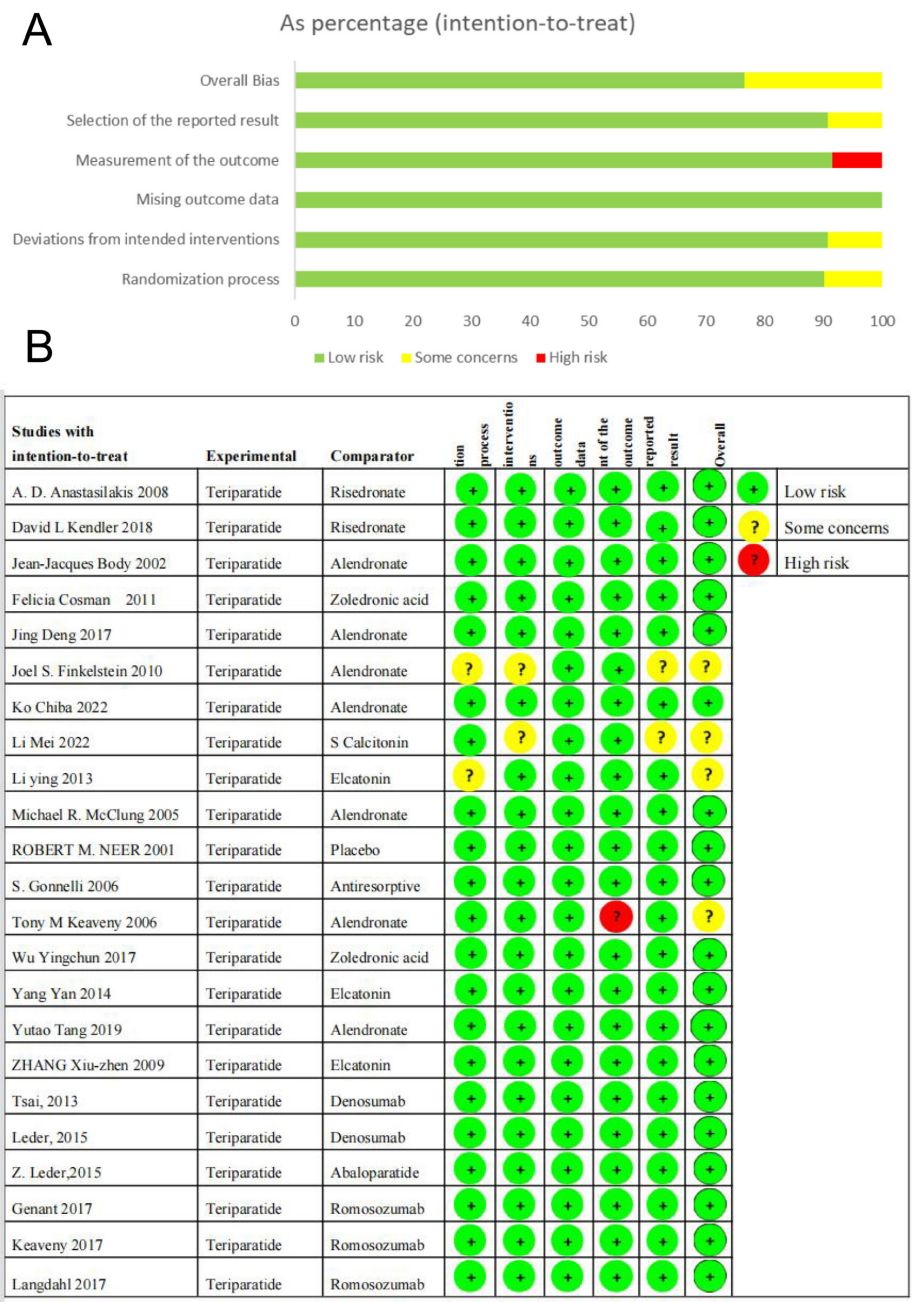
(A) A proportional risk of bias graph is presented, showcasing the percentage of biased items from all the studies included in the analysis (20, 50, 51, 52, 53, 54, 55, 56, 57, 58, 59, 60, 61, 62, 63, 64, 65, 66, 67, 68, 69, 70, 71). This visual representation allows for a clear judgment of the level of bias present in the studies and provides insight into the overall reliability of the results. (B) Diagram Illustrating Risk of Bias: Evaluating Bias in Various Aspects Across Included Studies (20, 50, 51, 52, 53, 54, 55, 56, 57, 58, 59, 60, 61, 62, 63, 64, 65, 66, 67, 68, 69, 70, 71).

We used Rob 2 2 (Fig. 2A and B) and the RevMan funnel plot to evaluate publication bias (See supplementary material). The results did not show any clear evidence of publication bias. We also performed Egger’s test to assess publication bias. Our analysis showed significant heterogeneity among the studies for continuous variable data. The *I* ^2^ value was 71.1%, and the estimate of between-study variance was Tau-squared = 0.3042. The OR = 1 test showed a significant difference in ORs across studies. Egger’s test revealed a non-statistically significant bias coefficient, indicating that the variations are not due to chance (See supplementary material).

Regarding binary variable data, our findings revealed significant heterogeneity among the studies, with an *I* ^2^ value of 99.0%. This suggests that factors other than chance may affect the results, and the estimate of between-study variance was Tau-squared = 7.6244. However, we found no significant difference in effect sizes across studies, indicating that the variations are not due to chance. Egger’s test exhibited no clear indication of publication bias.

In conclusion, while no publication bias was found for binary and continuous variable data, significant heterogeneity was present for binary variable data. Therefore, it is necessary to exercise caution while interpreting the results of this meta-analysis.

### Primary Outcome

#### Efficacy of teriparatide

##### Incidence of vertebral fracture:

An analysis of 11 studies involving 2756 patients was conducted to compare the incidence of fractures in the teriparatide-treated and the control groups. The studies had minimal heterogeneity (*I*^2^ = 31%, *P* = 0.16), indicating a consistent pattern of results, allowing for using a fixed-effects model to synthesize the data.

**Figure 3:**
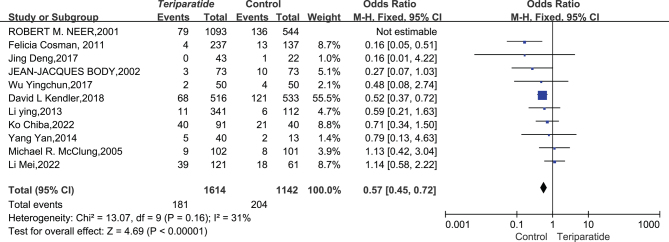
Forest plot comparing the incidence of fractures between teriparatide and control groups (20, 51, 52, 53, 54, 55, 57, 60, 61, 62, 64).

The synthesized outcomes showed a significant reduction in fracture risk in the teriparatide group compared to the control group, with a –relative risk (RR) of 0.57 and a 95% CI ranging from 0.45 to 0.72. These results were statistically significant (*P* = 0.00001), as shown in Fig. 3. These data confirm that teriparatide, also known as PTH 1–34, effectively reduces the likelihood of fracture events compared to a placebo, emphasizing its efficacy in fracture risk management in orthopedic patient care.

#### BMD

**Total hip BMD result**: After reviewing data from 12 studies with 3536 participants, we investigated the effect of teriparatide (PTH 1–34) injections on total hip BMD compared to a control group without treatment. Due to the high variability in study results (*I*² = 100%, *P* < 0.00001), a random-effects model was used for analysis.

**Figure 4:**
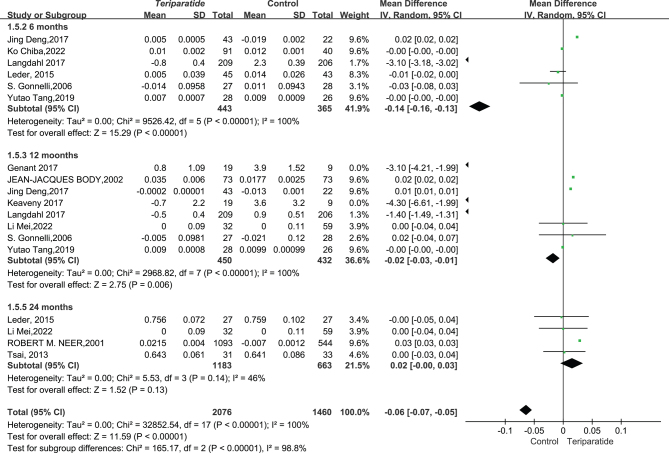
This forest plot diagram illustrates changes in bone mineral density between teriparatide and placebo treatments at the hip (52, 54, 55, 59, 61, 65, 67, 69, 70, 71, 72).

The results did not indicate a significant improvement in total hip BMD at 6, 12, and 24 months post treatment with teriparatide. The outcomes were as follows: at 6 months, the mean difference was slight (MD = −0.14, with 95% CI between −0.16 and −0.13); at 12 months, the improvement was minimal (MD = −0.02, with 95% CI between −0.03 and −0.01); and at 24 months, there was almost no change (MD = 0.02, with 95% CI between −0.00 and 0.03). The overall analysis, incorporating all time points, showed an overall mean difference of −0.06 (with 95% CI between −0.07 and −0.05), indicating that teriparatide did not have a significant impact on total hip BMD compared to controls, as depicted in Fig. 4.

In conclusion, this synthesis suggests that teriparatide may not have a marked effect on hip BMD, prompting clinicians to consider other factors or treatments in managing patients’ bone health.

#### Lumbar BMD result:

The study analyzed data from 20 articles involving 6356 patients to examine the effects of teriparatide (PTH) on lumbar BMD over time, comparing it to control groups. Due to diverse study outcomes (*I*² = 100%, *P* < 0.00001), a random-effects model was employed.

Initially, at 3 and 6 months, there was no significant change in lumbar BMD between the teriparatide and control groups, with MDs of 0.01 (95% CI: −0.02–0.04, *P* = 0.44) and 0.00 (95% CI: −0.01–0.01, *P* = 0.37) respectively. However, at 12 months, there was a significant decrease in BMD in the teriparatide group (MD = −0.07, 95% CI: −0.10 to −0.05, *P* < 0.00001), which shifted to significant increases at 18 months (MD = 0.05, 95% CI: 0.01–0.09, *P* = 0.01) and 24 months (MD = 0.05, 95% CI: 0.04–0.07, *P* < 0.00001).

**Figure 5:**
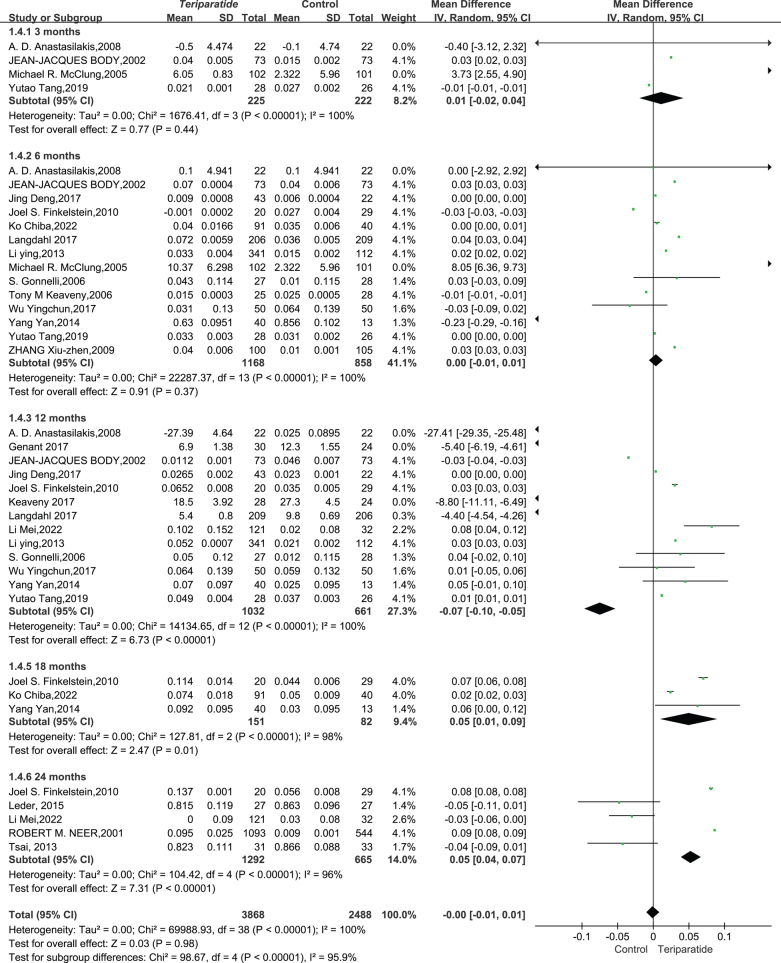
This forest plot diagram illustrates changes in bone mineral density between teriparatide and placebo treatments at the lumbar spine (50, 51, 52, 53, 54, 54, 56, 57, 59, 60, 61, 62, 63, 64, 65, 66, 68, 69, 70, 71).

Considering all time points, there was no significant long-term difference in lumbar spine BMD changes between the teriparatide treatment and control groups (MD = −0.00, 95% CI: −0.01–0.01, *P* = 0.98), as shown in Fig. 5, indicating that teriparatide’s benefits on lumbar BMD emerge over time, especially at 18 and 24 months, suggesting the importance of long-term treatment for bone density improvements.

#### Femoral neck BMD result:

We analyzed 15 studies with 5742 patients, focusing on the change in BMD at the femoral neck. Given the extensive variability in results across these studies (*I*² = 100%, *P* < 0.00001), a random-effects model was employed to synthesize the data accurately.

The analysis showed no significant differences in femoral neck BMD changes at 6 months (MD = −0.00, 95% CI = (−0.00, 0.00), *P* = 0.93), 12 months (MD = −0.01, 95% CI = (−0.02, −0.00), *P* = 0.01), and 18 months (MD = 0.00, 95% CI = (−0.02, 0.02), *P* = 0.97). However, significant changes were observed at 3 months (MD = 0.00, 95% CI = (0.00, 0.00), *P* < 0.0001) and particularly at 24 months (MD = 0.03, 95% CI = (0.03, 0.03), *I*² = 0%, *P* < 0.00001), indicating a notable improvement in the femoral neck BMD for the PTH 1–34 group compared to controls.

**Figure 6:**
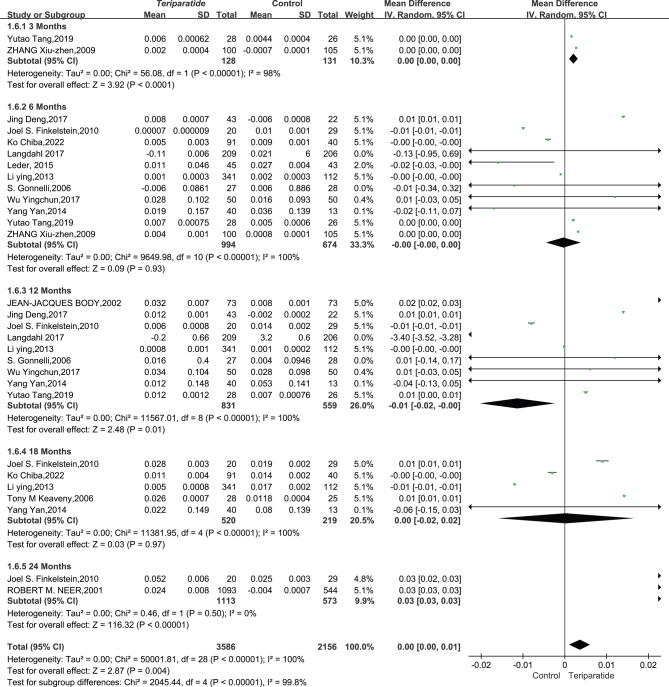
This forest plot diagram illustrates changes in bone mineral density between teriparatide and placebo treatments at the femoral neck (20, 53, 54, 55, 56, 59, 60, 62, 63, 64, 65, 68, 70, 71).

The overall data revealed a statistically significant improvement in femoral neck BMD (MD = 0.00, 95% CI = (0.00, 0.01), *I*² = 100%, *P* = 0.004). This underscores the significant positive effect of prolonged teriparatide treatment on femoral neck BMD, as depicted in Fig. 6. The results highlight the benefits of extended teriparatide administration for enhancing femoral neck BMD compared to control groups, indicating its efficacy in this specific aspect of patient care.

#### Radius BMD result

Data from two studies involving 2046 patients were analyzed to assess the impact of teriparatide (PTH 1–34) on radial BMD over time, compared to a control group. Due to the high degree of variability in results across these studies (*I*² = 100%, *P* < 0.00001), a random-effects model was applied for analysis to accommodate the observed statistical heterogeneity.

The analysis revealed that at 6, 18, and 24 months, there were no statistically significant changes in radial BMD between the Teriparatide and the control groups. Specifically, at 6 months, the mean difference (MD) was −0.01 (95% CI: −0.02–0.01, *I*² = 93%, *P* = 0.28), at 18 months, MD was −0.01 (95% CI: −0.06–0.03, *I*² = 100%, *P* = 0.63), and at 24 months, MD was −0.01 (95% CI: −0.08–0.05, *I*² = 100%, *P* = 0.72), indicating no significant change in radial BMD due to teriparatide treatment over these periods. The aggregated data from these time points showed an overall MD of −0.01 (95% CI: −0.02–0.01, *I*² = 100%, *P* = 0.23).

**Figure 7:**
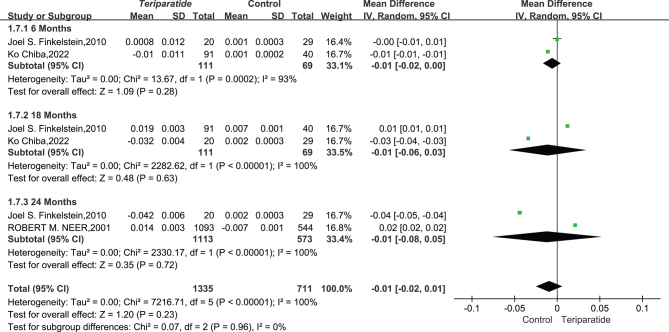
This forest plot diagram illustrates changes in bone mineral density between teriparatide and placebo treatments at the radial bone (20, 56, 60).

These findings, as illustrated in Fig. 7, suggest that long-term teriparatide treatment does not significantly affect radial BMD compared to controls. This outcome highlights the nuanced effectiveness of teriparatide, indicating that while it may have significant effects on other bone regions, its impact on radial BMD over the studied durations is minimal.

## Secondary outcome

### Bone turnover markers

#### Osteocalcin result:

Data from two key studies involving 206 participants were analyzed to examine the impact of teriparatide (PTH 1–34) on osteocalcin levels, a marker of bone formation. The studies exhibited a high degree of variability in their results (*I*² = 100%, *P* < 0.00001), necessitating using a random-effects model for synthesis.

At the 6-month evaluation, the difference in osteocalcin levels between the teriparatide group and the control group was not statistically significant, with a mean difference (MD) of 60.64 and a 95% CI ranging from −38.35 to 159.63 (*I*² = 100%, *P* = 0.23). However, a significant increase in osteocalcin levels was observed at 12 months in the Teriparatide group, with an MD of 100.82 and a 95% CI of 93.20–108.44 (*I*² = 0%, *P* < 0.00001).

**Figure 8:**
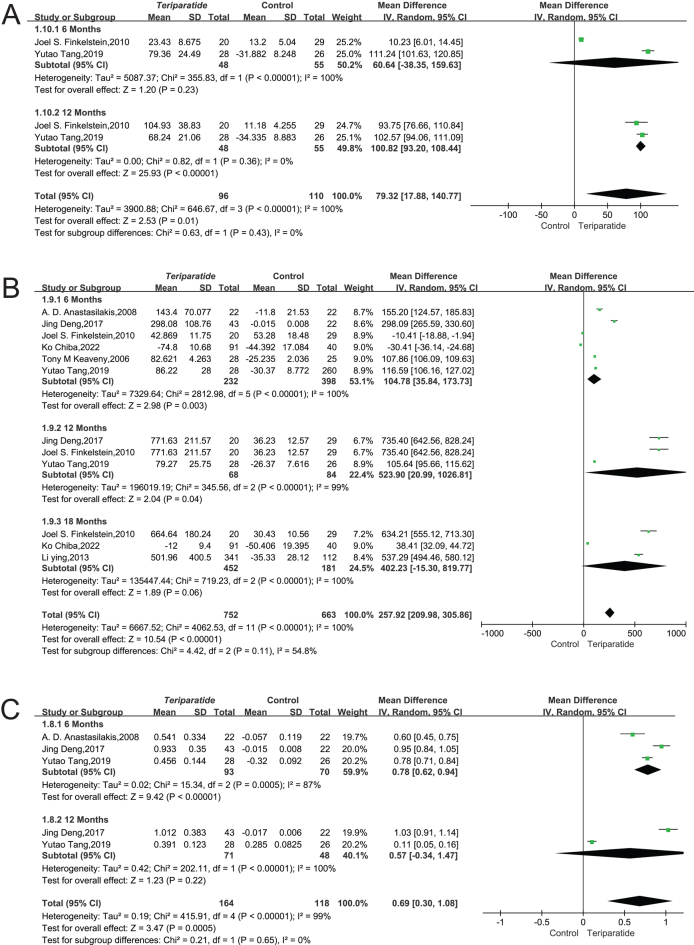
(A) Forest plot illustrating the effects of teriparatide and placebo treatments on osteocalcin at 6 and 12 months (56, 59). (B) Forest plot illustrating the effects of teriparatide and placebo treatments on P1NP at 6,12, and 18 months (50, 55, 56, 58, 59, 62). (C) Forest plot illustrating the effects of teriparatide and placebo treatments on CTx changes at 6 and 12 (50, 55, 59).

When considering the combined results across time points, the overall analysis indicated a significant enhancement in osteocalcin levels with an MD of 79.32 and a 95% CI of 17.88–140.77 (*I*² = 100%, *P* = 0.01), as depicted in Fig. 8. This suggests that long-term treatment with teriparatide significantly boosts osteocalcin levels in patients, demonstrating its effectiveness in enhancing bone metabolism compared to the control group.

#### P1NP result

Data from seven studies involving 1415 patients to assess changes in the biomarker P1NP, a marker of bone formation, following treatment with PTH (1–34) versus a control group. Due to high variability in the results across these studies (*I*² = 100%, *P* < 0.00001), a random-effects model was applied to accurately synthesize the findings.

The analysis revealed a significant difference in P1NP levels between the Teriparatide group and the control group at 6 and 12 months, with an MD of 104.78 and a 95% CI ranging from 35.84 to 173.73 (*I*² = 87%, *P* < 0.00001), indicating a substantial increase in bone formation in the treatment group. However, at the 18-month mark, the difference between groups was not statistically significant (MD = 402.23, 95% CI = −15.30–819.77, *I*² = 100%, *P* = 0.06), suggesting a variance in treatment response over time.

Overall, when considering the entire treatment period, the data showed a significant improvement in P1NP levels with an overall MD of 257.92 (95% CI = 209.98–305.86, *I*² = 100%, *P* < 0.00001) in the teriparatide-treated group compared to controls, as illustrated in Fig. 8B. This indicates that long-term Teriparatide administration significantly enhances bone formation, highlighting its efficacy in improving skeletal health over time.

#### C-telopeptide result

Data from three studies involving 282 participants were analyzed to evaluate the impact of teriparatide (PTH 1–34) on C-telopeptide levels, a marker of bone resorption. The substantial variability in results across these studies (*I*² = 100%, *P* < 0.00001) necessitated using a random-effects model for a comprehensive analysis.

At the 6-month interval, the analysis revealed a significant difference in C-telopeptide levels between the Teriparatide group and the control group, with a mean difference (MD) of 0.78 and a 95% CI from 0.62 to 0.94, indicating a pronounced reduction in bone resorption in the teriparatide group (*I*² = 87%, *P* < 0.00001). However, by the 12-month mark, this significant difference diminished (MD = 0.57, 95% CI = −0.34–1.47, *I*² = 100%, *P* = 0.22), suggesting a temporal effect of the treatment.

Overall, the aggregated data across the studies showed a significant reduction in C-telopeptide levels with teriparatide treatment over time (MD = 0.69, 95% CI = 0.30–1.08, *I*² = 99%, *P* < 0.00005), as presented in Fig. 8C. This indicates that long-term treatment with teriparatide significantly affects bone turnover markers, emphasizing its beneficial role in managing bone resorption in patients.

### Safety of teriparatide

#### Adverse events

The review analyzed 13 papers involving 4945 patients, focusing on the frequency of adverse events reported. The consistency across these studies was high, as indicated by the absence of statistical heterogeneity (*I*² = 0%, *P* = 0.77), which justified using a fixed-effects model for analysis.

**Figure 9:**
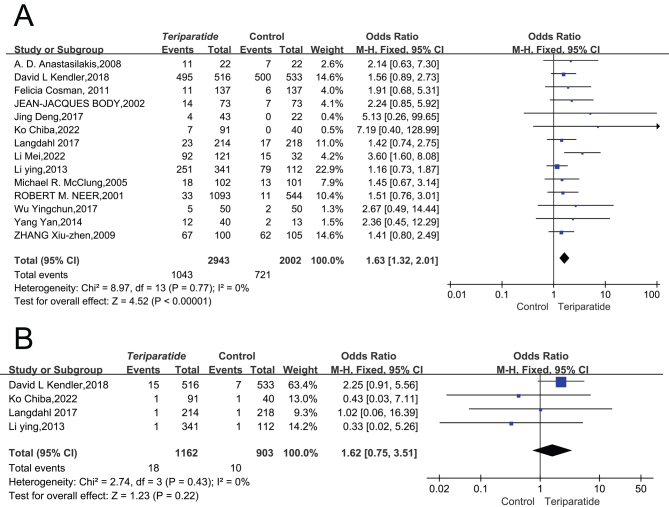
(A) Forest plot comparing the safety of teriparatide and placebo treatments in terms of adverse events (20, 50, 51, 52, 53, 54, 55, 56, 57, 60, 61, 62, 64). (B) Forest plot comparing the safety of teriparatide and placebo treatments in terms of Sudden death (20, 50, 51, 52, 53, 54, 55, 57, 60, 61, 62, 64).

The findings revealed that the group receiving the experimental treatment experienced more adverse events than the control groups, with an RR of 1.63 and a 95% CI of 1.32–2.01. This outcome suggests that the experimental group has a higher likelihood of adverse events than other control groups, as detailed in Fig. 9A. This analysis highlights the need for careful consideration of the safety profile of the experimental treatment in comparison to alternatives.

#### Death

Four publications, capturing 2065 patients, meticulously addressed the incidence of death across both cohorts. The statistical variation, *I*^2^ = 100.0%, signifies uniformity in the trials’ results, which supports applying a fixed-effects model to derive the combined metric. No noteworthy disparities were observed between the death rates of the two groups regarding patient safety (RR = 1.62, 95% CI: 0.75–3.51). Thus, juxtaposing the intervention group with the control group, the data suggest no significant enhancement in patient safety (Fig. 9B). The analysis indicates that there is no significant difference in the occurrence of death between the intervention group and the control group.

## Discussion

This meta-analysis sought to evaluate the effectiveness and safety of teriparatide (PTH 1–34) in comparison to alternative treatments or placebo in postmenopausal women diagnosed with osteoporosis. A thorough literature review was conducted across various databases from their inception until January 2023. Twenty-two studies were included to evaluate the effectiveness of teriparatide (PTH 1–34) compared to other treatments or placebo. The control groups in the trials comprised risedronate in two studies (50, 51), zoledronic acid in two (52, 53), alendronate in seven (54, 55, 56, 57, 58, 59, 60), salmon calcitonin in one (61), elcatonin in three (62, 63, 64), an antiresorptive agent in one (65), placebo in one (20), denosumab in two (66, 67), abaloparatide in one (68), and romosozumab in three (69, 70, 71). The research was conducted in various regions, including 7 studies in China, 11 in the USA, 1 each in Italy, Greece, and Japan, and 2 multicenter studies.

### Comparison with previous studies

Our study provides important insights into the treatment of postmenopausal osteoporosis. Our findings suggest that teriparatide is more effective than bisphosphonates in reducing fracture risk and improving lumbar spine BMD. This aligns with previous research, such as that conducted by Yuan *et al.* (72), which also demonstrated teriparatide’s efficacy in enhancing BMD.

Our analysis did not focus on fracture healing, but the positive effects of teriparatide on bone quality suggest that it may also benefit the fracture healing process. Studies have shown that teriparatide can improve bone density and reduce fracture risks, which could lead to better healing outcomes (29, 30, 31, 32, 33, 34). However, the studies we reviewed did not provide enough direct evidence on fracture healing rates, so further research is needed to fully understand teriparatide’s impact on this area.

A meta-analysis conducted by Liu *et al.* (73) demonstrated that romosozumab is highly effective in reducing the risk of various types of fractures and improving BMD in different regions compared to other therapies. Our study supports these findings and highlights the potential of romosozumab in managing osteoporosis. Another study suggested that combining teriparatide and denosumab may lead to better results in terms of BMD in the lumbar spine and hip compared to monotherapy (74). This indicates a promising direction for future treatment strategies and is consistent with our findings.

Simpson *et al.* (75) also highlighted the effectiveness of non-bisphosphonate treatments, including teriparatide, romosozumab, denosumab, and raloxifene, in preventing osteoporotic fragility fractures. Their findings support our conclusions, further emphasizing the importance of these drugs in the clinical landscape. Lastly, Hong *et al.* (76) conducted a meta-analysis comparing the efficacy of abaloparatide and teriparatide, with results suggesting a more pronounced increase in BMD with abaloparatide. While our study did not compare treatment modalities in detail, the findings of this study provide an important perspective on the evolving treatment methods.

Our study contributes to a better understanding of postmenopausal osteoporosis treatment. It is clear that different treatment modalities, either alone or in combination, have unique advantages that can be utilized for the best possible patient outcomes.

### Novel contributions and strengths of our meta-analysis

Our meta-analysis significantly enhances the understanding of teriparatide (PTH 1–34) therapy for treating postmenopausal osteoporosis, particularly highlighting its impact on radius bone mineral density (BMD) – an aspect that needs to be thoroughly explored. We provide an in-depth review of teriparatide’s effectiveness in increasing lumbar and femoral neck BMD, demonstrating its potential to reduce fracture risks and aid fracture healing in high-risk postmenopausal women. Our study also balances the benefits of teriparatide with its adverse effects, advocating for a nuanced approach to its clinical use. We pinpoint gaps in research like long-term safety and optimal treatment protocols, proposing directions for further investigation. By comparing teriparatide with other treatments and suggesting the exploration of new delivery methods, our analysis aims to innovate osteoporosis management, thereby enriching clinical decision-making and guiding future research.

### Limitations of our meta-analysis

Our meta-analysis, while methodologically rigorous and guideline compliant, faces several limitations. First, including studies from diverse countries introduces variability due to differing patient populations and treatment protocols. Secondly, there is noticeable heterogeneity within control groups. Thirdly, participants’ osteoporosis severity varied. Fourthly, a comprehensive understanding of the long-term effectiveness and reliability of teriparatide (PTH 1–34) treatment needs to be improved, particularly its impact on fracture healing. Fifthly, there were inconsistencies in teriparatide dosages among studies. Our innovative focus on radius BMD limited the analysis to a smaller subset of studies, possibly affecting the robustness of our conclusions.

Additionally, incorporating long-term data and fracture healing was challenging due to variable study lengths and follow-up quality. Lastly, despite a thorough assessment of adverse safety events, differences in reporting standards across studies may need to be clarified. Despite these limitations, our analysis provides valuable insights into treating postmenopausal osteoporosis with teriparatide (PTH 1–34).

## Future research directions

The current research highlights the need for further exploration into teriparatide (PTH 1–34) treatment for postmenopausal osteoporosis, particularly its long-term safety, effectiveness, and impact on fracture healing. Future studies should focus on conducting large-scale, well-designed, RCTs to understand the optimal dosage, treatment duration, and frequency. Comparing teriparatide’s efficacy with other interventions and investigating continuous versus intermittent therapy and new delivery methods are also crucial. Ensuring safety through detailed adverse event analysis, including mortality, is essential. While teriparatide presents a promising option for those at high fracture risk or unresponsive to other treatments, a personalized assessment of its benefits and risks, particularly regarding fracture healing, is necessary.

### Clinical implications of our study

Our study enhances the understanding of teriparatide (PTH 1–34) in managing postmenopausal osteoporosis, particularly highlighting its significant impact on the radius BMD and the lumbar and femoral neck regions. This insight is crucial for reducing vertebral fracture risks and influencing fracture healing. By demonstrating teriparatide’s dual action on bone metabolism, stimulating the formation, and potentially increasing resorption, our findings provide critical information for clinicians, especially regarding fracture healing.

It emphasizes the importance of personalized treatment strategies and careful patient monitoring, especially for long-term therapy, due to the observed rise in adverse events. Our research advocates for continued investigation into teriparatide’s long-term safety and efficacy, aiming for a more personalized, evidence-based clinical application that aligns with patient-specific needs and the latest scientific developments.

## Conclusion

Our study reaffirms the role of teriparatide (PTH 1–34) in treating postmenopausal osteoporosis, uniquely highlighting its efficacy not only in improving lumbar and femoral neck bone mineral density but also in enhancing radius BMD, a less explored yet significant aspect. This tripartite improvement in skeletal health, a novel contribution of our analysis, underscores teriparatide’s comprehensive potential in reducing fracture risks, particularly vertebral fractures. Despite its promising osteoanabolic action, the need for a balanced consideration of bone formation against potential resorption and the careful monitoring of adverse events is paramount. Our findings advocate for meticulous patient selection and ongoing safety evaluations, especially considering the long-term use of teriparatide. Moreover, our study calls for continued research into optimizing dosing strategies and evaluating teriparatide’s efficacy relative to emerging osteoporosis treatments. Our study positions teriparatide (PTH 1–34) as a key yet intricate therapy in osteoporosis management, emphasizing a personalized and evolving approach in its clinical application, informed by ongoing research and individual patient profiles.

## Supplementary Materials

Supplemental Material

Supplemental Figure

Supplementary Table

## ICMJE Conflict of Interest Statement

The authors declare that there is no conflict of interest that could be perceived as prejudicing the impartiality of the study reported.

## Funding Statement

This work was supported by National Key R&D Program of China (no. 2023YFC3603400), National Natural Science Foundation of Chinahttp://dx.doi.org/10.13039/501100001809 (no. 82072506, 82272611, and 92268115), Hunan Provincial Science Fund for Distinguished Young Scholars (no. 2024JJ2089), Science and Technology Innovation Program of Hunan Province (no. 2021RC3025 and 2023SK2024), Natural Science Foundation of Hunan Province (no. 2023JJ30949) and Natural Science Foundation of Shaanxi Provincehttp://dx.doi.org/10.13039/501100007128 (no. 2021JM-576).

## Availability of data and materials

The data supporting the findings of this study are included in this article.

### Author contribution statement

All authors have reviewed and agreed upon the manuscript. DTAV conceptualized the study, reviewed and edited the manuscript, handled the software, and created the original draft. AEE conducted data collection and reviewed the manuscript. MR contributed to the methodology and data curation. MO performed the formal analysis and investigation. DKY and RMG worked on visualization and investigation, while GFLS and AK contributed to the methodology and visualization. Lastly, XW and YL provided supervision, project administration, validation, and funding acquisition. The protocol can be accessed through PROSPERO (protocol ID: CRD42023392628).
