# Supplementary material for: Teriparatide in postmenopausal osteoporosis: uncovering novel insights into efficacy and safety compared to other treatments – a systematic review and meta-analysis
Source: EFORT Open Rev. 2024 Sep 2;9(9):845–61. doi: 10.1530/EOR-23-0205 (PMC11457814; doi:10.1530/EOR-23-0205)
Supplement: Supplemental Material [file EOR-23-0205supplementary_material.pdf]

Efficacy and Safety of Teriparatide Compared to Other Treatments for Postmenopausal Osteoporosis: A Systematic Review and Meta-Analysis of Randomized Controlled Trials

Pubmed search: from 2000- January 2023

(((((((("Parathyroid Hormone"[Mesh]) OR (· Hormone, Parathyroid" OR (· Parathormone" OR (· Parathyrin" OR (· Parathyroid Hormone Peptide (1-34") OR (· Parathyroid Hormone (1-34") OR (· PTH(1-34") OR (· Natpara" AND ((((((((((((((("Osteoporosis, Postmenopausal"[Mesh]) OR (· Perimenopausal Bone Loss" OR (· Bone Loss, Postmenopausal" OR (· Bone Losses, Postmenopausal" OR (· Postmenopausal Bone Losses" OR (· Osteoporosis, Post-Menopausal" OR (· Osteoporoses, Post-Menopausal" OR (· Osteoporosis, Post Menopausal" OR (· Post-Menopausal Osteoporoses" OR (· Post-Menopausal Osteoporosis" OR (· Postmenopausal Osteoporosis" OR (· Osteoporoses, Postmenopausal" OR (· Postmenopausal Osteoporoses" OR (· Bone Loss, Perimenopausal" OR (· Bone Losses, Perimenopausal" OR (· Perimenopausal Bone Losses" OR (· Postmenopausal Bone Loss") AND ("randomized controlled trial"[pt] OR "controlled clinical trial"[pt] OR randomized[tiab] OR placebo[tiab] OR "drug therapy"[sh] OR randomly[tiab] OR trial[tiab] OR groups[tiab]) AND (2000:2023[pdat]).

Web of science search: from 2000- January 2023

TS=("Parathyroid Hormone" OR "Hormone, Parathyroid" OR "Parathormone" OR "Parathyrin" OR "Parathyroid Hormone Peptide (1-34)" OR "Parathyroid Hormone (1-34)" OR "PTH(1-34)" OR "Natpara")
AND TS=("Osteoporosis, Postmenopausal" OR "Perimenopausal Bone Loss" OR "Bone Loss, Postmenopausal" OR "Bone Losses, Postmenopausal" OR "Postmenopausal Bone Losses" OR "Osteoporosis, Post-Menopausal" OR "Osteoporoses, Post-Menopausal" OR "Osteoporosis, Post Menopausal" OR "Post-Menopausal Osteoporoses" OR "Post-Menopausal Osteoporosis" OR "Postmenopausal Osteoporosis" OR "Osteoporoses, Postmenopausal" OR "Postmenopausal Osteoporoses" OR "Bone Loss, Perimenopausal" OR "Bone Losses, Perimenopausal" OR "Perimenopausal Bone Losses" OR "Postmenopausal Bone Loss")
AND TS=("randomized controlled trial" OR "controlled clinical trial" OR "randomized" OR "placebo" OR "drug therapy" OR "randomly" OR "trial" OR "groups")
AND PY=(2000-2023)

Embase search: from 2000- January 2023

(Parathyroid Hormone/ OR "Parathormone"/ OR "Parathyrin"/ OR "Parathyroid Hormone Peptide (1-34)"/ OR "Parathyroid Hormone (1-34)"/ OR "PTH(1-34)"/ OR "Natpara") AND (Osteoporosis, Postmenopausal/ OR "Perimenopausal Bone Loss"/ OR "Bone Loss, Postmenopausal"/ OR "Bone Losses, Postmenopausal"/ OR "Postmenopausal Bone Losses"/ OR "Osteoporosis, Post-Menopausal"/ OR "Osteoporoses, Postmenopausal"/ OR "Osteoporosis, Post Menopausal"/ OR "Post-Menopausal Osteoporoses"/ OR "Post-Menopausal Osteoporosis"/ OR "Postmenopausal Osteoporosis"/ OR "Osteoporoses, Postmenopausal"/ OR "Postmenopausal

Osteoporoses"/ OR "Bone Loss, Perimenopausal"/ OR "Bone Losses, Perimenopausal"/ OR "Perimenopausal Bone Losses"/ OR "Postmenopausal Bone Loss") AND (Randomized Controlled Trial/ OR "Controlled Clinical Trial"/ OR randomized:ti,ab OR placebo:ti,ab OR "Drug Therapy"/ OR randomly:ti,ab OR trial:ti,ab OR groups:ti,ab) AND (2000-2023/pd)
