# Supplementary material for: Teriparatide in postmenopausal osteoporosis: uncovering novel insights into efficacy and safety compared to other treatments – a systematic review and meta-analysis
Source: EFORT Open Rev. 2024 Sep 2;9(9):845–61. doi: 10.1530/EOR-23-0205 (PMC11457814; doi:10.1530/EOR-23-0205)
Supplement: Supplemental Figure [file EOR-23-0205supplementary_figure.pdf]

Supplementary Materials

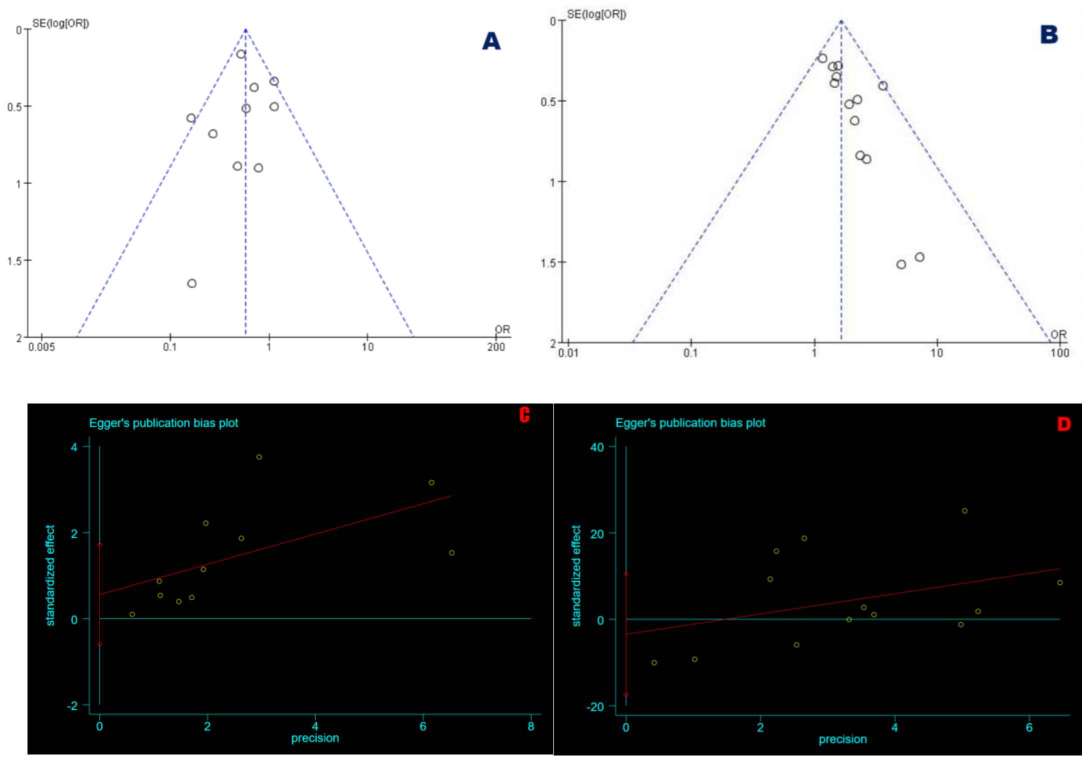

Figure 6. Funnel plots for (A)Adverse Event Incidence. (B) Fracture Incidence.

Egger's testfor (C) Continuous variable. (D) Binary variable
