# Supplementary material for: Teriparatide in postmenopausal osteoporosis: uncovering novel insights into efficacy and safety compared to other treatments – a systematic review and meta-analysis
Source: EFORT Open Rev. 2024 Sep 2;9(9):845–61. doi: 10.1530/EOR-23-0205 (PMC11457814; doi:10.1530/EOR-23-0205)
Supplement: Supplementary Table [file EOR-23-0205supplementary_table.pdf]

Supplementary Table 1: Summary of Characteristics for Included Studies in the Meta-Analysis

Authors, Years	Country	Sample size		Age, (Mean SD),		Adverse Event		Fracture		BMI (kg/m²; SD)		Skeletal site of BMD	Intervention		Follow-up (month)	BMD assessment
		PTH	Con	PTH	Con	PTH	Con	PTH	Con	PTH	Con	CT_X, P1NP,LS	PTH (μg/day)	Con		
Anastasilakis, 2008 (1)	Greece	22	22	65.1 ± 1.6	64.7 ± 1.5	11	7	NR	NR	28.2 ± 1.	28.1 ± 1.		20	risedronate 35mg/week	3,6 ,12	DXA
Kendler, 2018 (2)	Europe, South America, North America	516	533	72.6±8.77	71.6 ±8.58	495	500	68	121	26.9 ±4.61	27.1 ±4.64	NR	20	risedronate 20 μg/day	24	DXA
Felicia, Cosman, 2011 (3)	USA	137	137	65.0±8.8	66.1±9.0	11	6	4	13	25.3±4.15	25.3 ±4.42	CT_X, P1NP,LS, TH	20	zoledronic acid 5 mg	13	DXA
Yingchun, 2017 (4)	China	50	50	54.2 ± 8.2	54.2 ± 8.2	5	2	2	4	NR	NR	NR,LS ,FN	20	zoledronic acid 5 mg/day	6,12	DXA
Body, 2002 (5)	United States, Austria, Belgium, Canada, Israel, Mexico	73	73	66±8	65±9	14	7	3	10	23.9±4.5	24.4±3.5	LS, TH,FN	40	alendronate 10g /day	3,6,12,14	DXA
Deng, 2017 (6)	China	43	22	62.88±5.80	62.77±6.42	4	0	0	1	24.25±2.60	23.5±3.24	CT_X, P1NP,LS, TH,FN	20	alendronate 70mg/ week	6,12	DXA
Finkelstein, 2010 (7)	USA	20	29	65±7	64±6	NR	NR	NR	NR	25.6±4.5	23.4 ± 3.4	P1NP, Os,LS, FN,R	40	alendronate	3,6,18,24, 30	DXA

														10 mg/da		
Michael. McClung, 2005 (8)	USA	102	101	65.3 ± 8.4	66.6 ± 8.5	18	13	9	8	25.7 ± 4.0	25.3 ± 4.5	P1NP,LS, FN	20	Alendronate 35 mg/day	3,6,18	DXA
Keaveny, 2006 (9)	USA	28	25	64.5 ± 1.4	62.5 ± 1.6	NR	NR	NR	NR	26.3 ± 0.8	26.6 ± 0.9	CT_X, P1NP,LS ,FN	20	Alendronate 10 mg/day	6,18	DXA
Tang, 2019 (10)	China	28	26	61.7±6.6	62.8±7.3	NR	NR	NR	NR	24.8±1.3	26±4	CTX, P1NP,Os,LS, TH,FN,R	20	Alendronate 70mg/week	1,3,6,12	DXA
Chiba, 2022 (11)	Japan	91	40	71.7 ± 6.9	71.9 ± 7.0	7	0	40	21	23.4 ± 3.4	22.6 ± 2.9	P1NP,LS, TH,FN,R	20	Alendronate 35mg/week	6,18,	DXA
Mei, 2022 (12)	China	121	32	66.48±8.17	65.13±6.97	92	15	39	18	NR	NR	CT_X, P1NP,LS, FN	20	S. Calcitonin NR	12, 24	RCT
Li ying,2013 (13)	China	341	112	65.1±7.1	65.0±7.3	251	79	11	6	23.1 ± 3.1	23.4 ± 3.3	CT_X,LS, FN	20	Elcatonin 20U/week	6,12,18	DXA
Zhang, 2009 (14)	China	100	105	64.70±7.67	65.78±7.62	67	62	NR	NR	23.38±3.17	23.60±3.29	NR,LS ,FN	20	Elcatonin 20U/wks	3,6	DXA
Yan, 2014 (15)	China	40	13	65.1±7.6	65.5±8.7	12	2	5	2	NR	NR	CT_X,LS ,FN	20	Elcatonin 20U/week	6,12,18	DXA
Gonnelli, 2006 (16)	Italy	27	28	71.3±7.0	71.0±6.8	NR	NR	NR	NR	24.7±3.3	24.8±3.1	LS, TH,FN	20	antiresorptive agent NR	6,12	DXA
Neer, 2001 (17)	USA	1093	544	69±7	69±7	33	11	79	136	26.6±4.3	26.7±4.7	LS, TH,FN,R	20	Placebo NR	24	DXA
Tsai, 2013(18)	USA	27	23	65.7±7.9	66.3±8.3	1	1	NR	NR	25.5±3.8	24.9±3.9	LS, TH	20	Denosumab 60 mg /6 months	24	DXA

Leder, 2015(19)	USA	27	27	66.1±7.9	65.0±6.2	6	4	14	10	25.5±3.7	23.8±4.1	TH	20	Denosumab 60 mg /6 months	24	DXA
Z. Leder 2015 (20)	USA	45	43	64.5±7.5	66.3±7.0	12	9	NR	NR	26.0±3.4	26.4 ±2.9	TH,FN	20	Abaloparatide 20/d	6,12	DXA
Genant 2017(21)	USA	31	24	65.8±5.7	64.3±4.7	NR	NR	NR	NR	NR	NR	LS, TH, FN	20	Romosozumab 210 mg / month	12	DXA
Keaveny 2017(22)	USA	31	24	65.8±5.7	64.3±4.7	NR	NR	NR	NR	NR	NR	LS	20	Romosozumab 210 mg / month	12	DXA
Langdahl 2017 (23)	USA	214	218	71.2±7.7	71.8±7.4	148	164	NR	NR	NR	NR	TH	20	Romosozumab 210 mg / month	6,12	DXA

PTH – parathyroid hormone；Con – Control；NR– Not reported; BMD – bone mineral density; SI – subcutaneous injection; LS – lumbar spine; TH – total hip;FN –Femoral Neck; R – radius; DXA – dual-energy；x-ray– absorptiometry; CTx – xbeta-C-terminal telopeptide ; P1NP – type 1 procollagen amino-terminal peptides ;Os – osteocalcin.

Reference

[1].Anastasilakis, A. D., Goulis, D. G., Polyzos, S. A., Gerou, S., Koukoulis, G. N., Efstathiadou, Z., ... & Avramidis, A. (2008). Head-to-head comparison of risedronate vs. teriparatide on bone turnover markers in women with postmenopausal osteoporosis: a randomized

trial. International journal of clinical practice, 62(6), 919-924.

[2].Kendler, D. L., Marin, F., Zerbini, C. A., Russo, L. A., Greenspan, S. L., Zikan, V., ... & López-Romero, P. (2018). Effects of teriparatide and risedronate on new fractures in postmenopausal women with severe osteoporosis (VERO): a multicentre, double-blind, double-dummy, randomized controlled trial. The Lancet, 391(10117), 230-240.

[3].Cosman, F., Eriksen, E. F., Recknor, C., Miller, P. D., Guañabens, N., Kasperk, C., ... & Boonen, S. (2011). Effects of intravenous zoledronic acid plus subcutaneous teriparatide [rhPTH (1–34)] in postmenopausal osteoporosis. Journal of Bone and Mineral Research, 26(3), 503-511.

[4].Wu Yingchun, Sun Hui. Comparative study of the efficacy of parathyroid hormone (1-34), strontium ranelate and zoledronic acid in postmenopausal osteoporosis [J]. Chinese Journal of Osteoporosis, 2017,23 (10): 1276-1279.

[5].Body, J. J., Gaich, G. A., Scheele, W. H., Kulkarni, P. M., Miller, P. D., Peretz, A., ... & Hodsman, A. B. (2002). A randomized double-blind trial to compare the efficacy of teriparatide [recombinant human parathyroid hormone (1–34)] with alendronate in postmenopausal women with osteoporosis. The Journal of Clinical Endocrinology & Metabolism, 87(10), 4528-4535.

[6].Deng, J., Feng, Z., Li, Y., Pan, T., Li, Q., & Zhao, C. (2018). Efficacy and safety of recombinant human parathyroid hormone (1–34) are similar to those of alendronate in the treatment of postmenopausal osteoporosis. Medicine, 97(47).

[7].Finkelstein, J. S., Wyland, J. J., Lee, H., & Neer, R. M. (2010). Effects of teriparatide, alendronate, or both in women with postmenopausal osteoporosis. The Journal of Clinical Endocrinology & Metabolism, 95(4), 1838-1845.

[8].McClung, M. R., San Martin, J., Miller, P. D., Civitelli, R., Bandeira, F., Omizo, M., ... & Eriksen, E. F. (2005). Opposite bone remodeling effects of teriparatide and alendronate in increasing bone mass. Archives of internal medicine, 165(15), 1762-1768.

[9].Keaveny, T. M., Donley, D. W., Hoffmann, P. F., Mitlak, B. H., Glass, E. V., & San Martin, J. A. (2007). Effects of teriparatide and alendronate on vertebral strength as assessed by finite element modeling of QCT scans in women with osteoporosis. Journal of bone and mineral research, 22(1), 149-157.

[10].Tang, Y., Xia, H., Kang, L., Sun, Q., Su, Z., Hao, C., & Xue, Y. (2019). Effects of intermittent parathyroid hormone 1–34 administration on circulating mesenchymal stem cells in postmenopausal osteoporotic women. Medical Science Monitor: International Medical Journal of Experimental and Clinical Research, 25, 259.

[11].Chiba, K., Okazaki, N., Kurogi, A., Watanabe, T., Mori, A., Suzuki, N., ... & Osaki, M. (2022). Randomized controlled trial of daily teriparatide, weekly high-dose teriparatide, or bisphosphonate in patients with postmenopausal osteoporosis: The TERABIT study. Bone, 160, 116416.

[12].Li Mei, Zhang Zhenlin, Lin Hua, etc. Efficacy and safety of domestic rhPTH1-34 in treating postmenopausal osteoporosis: a randomized controlled, multicenter clinical study [J]. Chinese Journal of Osteoporosis and Bone Mineral Salt Diseases, 2022,15 (02): 135-141.

[13].Li, Y., Xuan, M., Wang, B., Yang, J., Zhang, H., Zhang, X. Z., & Guo, X. H. (2013). Comparison of parathyroid hormone (1-34) and elcatonin in postmenopausal women with osteoporosis: an 18-month randomized, multicenter controlled trial in China. Chinese Medical

Journal, 126(03), 457-463.

[14].Zhang, X. Z., Wang, B., Yang, J., Xuan, M., Song, L. G., Li, H., ... & JIA, Y. (2009). A randomized, multicenter controlled trial to compare the efficacy of recombinant human parathyroid hormone (1-34) with elcatonin in postmenopausal women with osteoporosis in China. Chinese Medical Journal, 122(24), 2933-2938.

[15].Yang Yan, Li Pengqiu, Zhu Xianjun, etc. Randomized controlled study of recombinant human parathyroid hormone (1-34) and descalcitonin for the treatment of primary osteoporosis [J]. Chinese Journal of Osteoporosis, 2014,20 (02): 137-141 + 155.

[16].Gonnelli, S., Martini, G., Caffarelli, C., Salvadori, S., Cadirni, A., Montagnani, A., & Nuti, R. (2006). Teriparatide's effects on quantitative ultrasound parameters and bone density in women with established osteoporosis. Osteoporosis International, 17, 1524-1531.

[17].Neer, R. M., Arnaud, C. D., Zanchetta, J. R., Prince, R., Gaich, G. A., Reginster, J. Y., ... & Mitlak, B. H. (2001). Effect of parathyroid hormone (1-34) on fractures and bone mineral density in postmenopausal women with osteoporosis. New England journal of medicine, 344(19), 1434-1441.

[18].Tsai, J. N., Uihlein, A. V., Lee, H., Kumbhani, R., Siwila-Sackman, E., McKay, E. A., et al. (2013). Teriparatide and Denosumab, Alone or Combined, in Women with Postmenopausal Osteoporosis: the DATA Study Randomised Trial. Lancet 382 (9886), 50–56. doi:10.1016/s0140-6736(13)60856-9

[19].Leder, B. Z., Tsai, J. N., Uihlein, A. V., Wallace, P. M., Lee, H., Neer, R. M., et al. (2015). Denosumab and Teriparatide Transitions in

Postmenopausal Osteoporosis (The DATA-Switch Study): Extension of a Randomised Controlled Trial. Lancet 386 (9999), 1147–1155. doi:10.1016/s0140-6736(15)61120-5

[20].Leder, Benjamin Z., et al. "Effects of abaloparatide, a human parathyroid hormone-related peptide analog, on bone mineral density in postmenopausal women with osteoporosis." The Journal of Clinical Endocrinology & Metabolism 100.2 (2015): 697-706.https://doi.org/10.1210/jc.2014-3718

[21].Genant HK, Engelke K, Bolognese MA, et al. Effects of romosozumab compared with teriparatide on bone density and mass at the spine and hip in postmenopausal women with low bone mass. J Bone Miner Res., 2017, 32: 181–187.https://doi.org/10.1002/jbmr.2932

[22].Keaveny TM, Crittenden DB, Bolognese MA, et al. Greater gains in spine and hip strength for Romosozumab compared with Teriparatide in postmenopausal women with low bone mass. J Bone Miner Res., 2017, 32: 1956–1962.https://doi.org/10.1002/jbmr.3176

[23].Langdahl BL, Libanati C, Crittenden DB, et al. Romosozumab (sclerostin monoclonal antibody) versus teriparatide in postmenopausal women with osteoporosis transitioning from oral bisphosphonate therapy: a randomised, open-label, phase 3 trial. Lancet., 2017, 390: 1585–1594.https://doi.org/10.1016/S0140-6736(17)31613-6
